# Supplementary material for: A structural equation model to access the regional public brands of agricultural products: Case of Chinese Yingde black tea
Source: PLoS One. 2024 Sep 27;19(9):e0310722. doi: 10.1371/journal.pone.0310722 (PMC11432859; doi:10.1371/journal.pone.0310722)
Supplement: S1 Appendix — (DOCX) [file pone.0310722.s001.docx]

**Appendix 1 Survey Questionnaire**

**农产品区域公用品牌形成机理调查问卷**

尊敬的先生/女士：

您好！本次调查目的在于研究农产品区域公用品牌形成机理，为英德红茶区域公用品牌的建设提供建议。请您根据实际情况回答，感谢您对本研究的支持和协助！

1.您的性别是（ ）

（1）男 （2）女

2.您了解广东英德红茶区域公用品牌吗？（ ）

（1）了解 （2）不了解（如果选择此项，以下题项不需要再填写。）

3.您的职业是（ ）

（1）政府工作人员

（2）英德红茶相关企业的从业人员

（3）英德红茶种植户

（4）英德红茶相关的研究人员

（5）英德红茶相关行业协会工作人员

（6）其他

4.您从事现有工作时间（ ）

（1）1-5年

（2）6-10年

（3）11-15年

（4）16-20年

（5）20年以上

以下是一些描述英德红茶区域公用品牌的语句。请根据您的认同程度进行选择，1-7分别代表：非常反对、反对、比较反对、中立、比较赞成、赞成、非常赞成。在符合 您观点的分数上画“√”。

| 每个题项的1-5分值表示从不同意到同意的渐进变化，请根据自身实际情况在相应的框内打√（1为非常反对，2为反对，3为比较反对，4为中立，5为比较赞成，6赞成，7非常赞成）。 | 非常反对⟷ 非常赞成 | | | | | | |
| --- | --- | --- | --- | --- | --- | --- | --- |
| **政府支持** | **1** | **2** | **3** | **4** | **5** | **6** | **7** |
| PS1.英德政府有颁布扶持英德红茶产业发展的政策。 |  |  |  |  |  |  |  |
| PS2.英德政府有投入财政资金扶持英德红茶产业发展。 |  |  |  |  |  |  |  |
| PS3.英德政府有加强红茶市场的监督和保护。 |  |  |  |  |  |  |  |
| PS4.英德政府有制定红茶产业发展的战略规划。 |  |  |  |  |  |  |  |
| PS5.英德政府有加大公共服务和设施的建设。 |  |  |  |  |  |  |  |
| **资源禀赋** | **1** | **2** | **3** | **4** | **5** | **6** | **7** |
| RE1.英德地区具有适合红茶种植的独特地理条件。 |  |  |  |  |  |  |  |
| RE2.英德地区具有独特的红茶品种。 |  |  |  |  |  |  |  |
| RE3.英德地区根据气象指数科学规划了红茶生产区域。 |  |  |  |  |  |  |  |
| RE4.英德地区发展了科学的红茶种植技术。 |  |  |  |  |  |  |  |
| **产业集群** | **1** | **2** | **3** | **4** | **5** | **6** | **7** |
| IC1.英德地区具有完整的红茶产业链。 |  |  |  |  |  |  |  |
| IC2.英德地区具有多元化的红茶产业结构。 |  |  |  |  |  |  |  |
| IC3.英德地区红茶相关企业之间有开展物流配送合作。 |  |  |  |  |  |  |  |
| IC4.英德地区红茶相关企业之间有开展技术创新合作。 |  |  |  |  |  |  |  |
| IC5.英德地区红茶相关企业之间有开展营销沟通与合作。 |  |  |  |  |  |  |  |
| **区域文化** | **1** | **2** | **3** | **4** | **5** | **6** | **7** |
| RC1.英德地区具有独特的红茶饮食文化。 |  |  |  |  |  |  |  |
| RC2.英德地区具有悠久的红茶种植文化。 |  |  |  |  |  |  |  |
| RC3.英德地区经常举办特色的红茶文化活动。 |  |  |  |  |  |  |  |
| RC4.英德地区将红茶文化融入了城市建设。 |  |  |  |  |  |  |  |
| **品牌美誉度** | **1** | **2** | **3** | **4** | **5** | **6** | **7** |
| BR1.英德红茶具有很高的品牌知名度。 |  |  |  |  |  |  |  |
| BR2.英德红茶具有独特的品牌形象。 |  |  |  |  |  |  |  |
| BR3.英德红茶具有优质的品牌口碑。 |  |  |  |  |  |  |  |
| BR4.英德红茶具有很多忠实的顾客重复购买。 |  |  |  |  |  |  |  |
