# Supplementary material for: A structural equation model to access the regional public brands of agricultural products: Case of Chinese Yingde black tea
Source: PLoS One. 2024 Sep 27;19(9):e0310722. doi: 10.1371/journal.pone.0310722 (PMC11432859; doi:10.1371/journal.pone.0310722)
Supplement: Attachment — Submitted filename: renamed_d8f0c.doc [file pone.0310722.s003.doc]

**Response to Reviewers**

Dear judges and experts:

Thank you very much for your revision suggestions. These revision suggestions can be very effective in helping me improve the quality of my paper. I have carefully revised the paper based on your revision suggestions. The following are specific modifications.

| **Expert serial number** | **Suggestion** | **Modification status** |
| --- | --- | --- |
| Reviewer #1 | 1. The model in Figure 1 is not an empirical model. It is a theoretical framework. That is, is a theory of potential drivers that represents the phenomenon under study. The empirical model is the statistical version of the theoretical framework. In this work, the empirical model is presented in Figure 2. | Change the "empirical model" in Figure 1 to "theoretical framework". |
|  | 2. I am very surprised to find that the authors did not include a table with the items that form part of the constructs. It is difficult to evaluate the meaning of the constructs without this information. Therefore, I require the authors to include the items considered in the questionnaire. An example of how a table of this nature can be included is presented in Tables 1 y 2 in May et al. (2021). This reference is listed below. | Thank you very much for recommending the reference paper to me.  I originally considered that the project table had a lot of content and was difficult to layout, so I submitted the measurement table as an attachment to the journal.But as you said, the problem this brings is that readers find it difficult to evaluate the significance of the scale structure.Therefore, I modified Table 1 to present the specific content of the scale structure.A survey questionnaire is also attached at the end of the paper. |
|  | 3. The constructs of the theoretical model are well justified from the literature. However, many works that use structural equation modelling in other contexts have also consider socio-psychological drivers. I think this is relevant for this research because farmers’ willingness to produce Yingde Black tea may also be influenced by these considerations (influence from the opinion of people in the social network such as friend, colleagues, family members, etc.; need for status, etc). I suggest the following examples to explain the relevance of socio-psychological considerations in decision making, and why they have been ignored in this draft:  Deng, J. Sun, P., Zhao, F., Hana, X., Yang, G. and Feng, Y. (2016). Analysis of the ecological conservation behavior of farmers in payment for ecosystem service programs in eco-environmentally fragile areas using social psychology models. Science of The Total Environment 550: 382-390.  May, D., Arancibia, S. and Manning, L. (2021). Understanding UK farmers’ Brexit voting decision: A behavioural approach. Journal of Rural Studies 81: 281-293.  May, D.E., Arancibia, S., Wang, C., Hill, N., and Behrendt, K. (2023). Understanding young Chinese consumers’ preferences for foreign clothing brands: a behavioural approach. Asia Pacific Journal of Marketing and Logistics 35(12): 3032-3051. | Your suggestion is correct. Many times, the willingness of farmers to grow Yingde black tea may also be influenced by these factors (opinions from people on social networks such as friends, colleagues, family, etc.; demands for status, etc.). However, this article focuses on the construction of regional public brands for agricultural products, involving the organizational level and not delving into the psychological level of farmers.So for now, social and psychological factors will not be included in the variables of structural equations in this study.  Studying regional public brands of agricultural products from a socio psychological perspective would be a very good choice, and therefore will be an important topic for our further in-depth research.We will conduct new research based on the research findings of Deng, J. (2016).This is presented in the future outlook of this article.  The research results of Deng, J. (2016), May, D. (2021), and May, D.E. (2023) have provided great inspiration for this study, providing both theoretical guidance and methodological demonstration.Therefore, all three papers have been included in the reference list in this article. |
|  | 4. The discussion can be enriched/improved by explaining the significant items that form part of the constructs. This has the potential to explore additional strategies to affect the regional public brand of agricultural products. An example of how the “empirical” model can be presented is shown in Figure 3 in May et al. (2021), listed above. | Referring to Figure 3 of May et al. (2021), an "empirical" model was created in the discussion section of the paper. |
|  | 5. Two very minor points. References in the main body should not include the name initials. For example, the authors wrote (Kavaratzis M., 2006), and should be (Kavaratzis, 2006). On the other hand, the authors list some shortcomings at the end of Section 2.1. The ideas listed in this case should be separated by ; (semicolons) (see Page 3). Please check the grammar in the full document. | Thank you very much for your careful review. There are indeed issues with these areas.Therefore, we have made the following modifications.  (1) The citation format has been modified according to the requirements of the journal.  (2) Use the separator ";" to separate the shortcomings of the parallel relationship.  (3) A consistency check was conducted on the professional terminology and citation style throughout the entire text, and modifications were made according to the requirements of the journal. |
| Reviewer #2 | Introduction: The introduction provides a clear overview of the significance of regional public brands for agricultural products and the specific focus on Chinese Yingde black tea. However, it could benefit from more precise delineation of the research objectives and hypotheses. Add more recent and relevant studies. | The following content has been added in the third paragraph of the introduction to clarify the research objectives of this article.  However, there has been a lack of research on the formation mechanism of regional public brands for agricultural products, as well as a lack of research on regional public brands for Chinese agricultural products. Therefore, this article will take Chinese Yingde black tea as the research object, explore the formation mechanism of regional public brands for agricultural products, study the influencing factors and interrelationships of regional public brands for agricultural products, and provide suggestions for building regional public brands for agricultural products based on this. |
|  | Literature Review: While the literature review briefly mentions the four main perspectives on the formation mechanism of regional public brands, it lacks depth and critical analysis of existing research. Consider expanding this section to include a more thorough synthesis of relevant literature, highlighting gaps and inconsistencies that the current study aims to address. | The literature review section has added recent relevant research literature from the four influencing factors of regional public brand construction, making the relevant literature more comprehensive and analyzing the controversies among different scholars, emphasizing the urgent issues and inconsistent viewpoints that need to be addressed in current research. |
|  | Methodology: The methodology section should provide more details regarding the survey questionnaire design, sampling strategy, and data collection process to ensure transparency and replicability of the study. | The survey questionnaire was submitted to the journal in the form of an appendix, which may have affected reading. This issue is indeed not conducive to readers understanding the entire text. Therefore, Table 1 has been modified to fully present the specific content of the scale structure.At the end of the paper, a survey questionnaire was also attached, and the original data was uploaded to the public data platform: Mendeley Data. The data's doi is 10.17632/9sys2yrwnp.1. URL of data: https://data.mendeley.com/drafts/9sys2yrwnp . These data can be obtained for free. |
|  | Clarify the rationale behind the choice of structural equation modeling (SEM) as the analytical technique and provide justification for the model specifications. | Add the following content to the "3.3. Data analysis" section to illustrate the basis for selecting a structural equation model.  Structural equation modeling is a multivariate statistical method based on variable covariance matrix for factor analysis and path analysis. It is mainly used to explore the potential relationships between multiple variables and comprehensively analyze the interaction mechanisms between variables (Tu Qihan, 2024). This article studies the formation mechanism of regional public brands for agricultural products, exploring the interaction between government support, resource endowment, industrial clusters, regional culture, and regional public brands for agricultural products.  Explain the normative requirements of the model in the text through the following content.  This article also conducted confirmatory factor analysis using AMOS 24 software. The smaller the ratio of chi square degrees of freedom(χ²/df), the higher the model's adaptability. Usually, a value less than 3 indicates good adaptability (Wen-e et al., 2021). When the root mean square error (RMSEA) of approximation is less than 0.08, it indicates good adaptation (Wen-e et al., 2021). The closer other indicators such as Comparative Fit Index (CFI) and Relative Fit Index (TLI) are to 1, the better the fit of the model. A value greater than 0.9 indicates better fit (Wen-e et al., 2021). |
|  | Conclusion: The conclusion should succinctly summarize the key findings, reiterate the study's contributions, and offer practical implications for stakeholders in real-world. Avoid introducing new information or ideas in the conclusion section, and focus on synthesizing the main insights derived from the study. | Thank you very much for your suggestion. The conclusion and theoretical contribution sections have been revised to provide a clearer and more explicit expression of the theoretical contribution of this article, and are presented separately in the form of 6.2. This can make it easy for readers to understand the theoretical contribution of this article. |
|  | Minor Comments: Ensure consistency in terminology and citation style throughout the manuscript. Proofread the manuscript for grammatical errors, typographical mistakes, and clarity of expression. | Thank you very much for your careful review. As the original manuscript of this article was written in Chinese, there were some confusion and even errors in the translation of professional terms into English, such as "scale" and "survey questionnaire", "government support" and "policy support". This revision has unified these professional terms. All scales have been changed to survey questionnaires, and all policy support has been changed to government support. |
| Reviewer #3 | 1. Authors' surnames and given names were misused for some papers, such as Juan & Jin 2022a, Yueli & Qiner, 2022, Yuhan et al., 2023, etc. Please double check the references. | This article has made revisions to the references and formatting requirements of PLOS one, as well as the format of the latest paper published in this journal.  Mockel S (2024) The macroeconomic money-nature nexus: Are growing money supplies a relevant obstacle on the way to an ecologically sustainable global economy? PLOS Sustain Transform 3(1): e0000095. <https://doi.org/10.1371/journal.pstr.0000095> |
|  | 2. In Introduction, a clear description of research gap could improve the article's strength as well as offer novelty. Are there really no comprehensive research about different factors of regional public brands? Why to choose these four factors? | This suggestion is very helpful for this article.  This revision adds recent relevant research literature in the literature review section from the four influencing factors of regional public brand construction, making the relevant literature more comprehensive. It also focuses on analyzing the controversies among different scholars, emphasizing the urgent issues and inconsistencies in viewpoints that need to be addressed in current research. This reflects the research value and novelty of this article. |
|  | 3. To evaluate the effectiveness of regional public brand construction for agriculture products, the degree of popularity or acceptence should also be considered besides brand repution. Otherwise, it is not successful for agricultural products since there may be a condition that people who know the brand think it's great, but only a few people know the brand. Evenmore, people think it's a good brand, but they don't want to buy. | Your viewpoint is correct. The effectiveness of brand building requires evaluating the level of customer recognition, identification, and acceptance.  The selection of brand reputation as the benchmark variable in this article is also based on this consideration. In the survey questionnaire, the measurement of brand reputation was set as brand awareness, brand image, brand evaluation, and customer repeat purchases.Perhaps the submission of the survey questionnaire in the form of an appendix to the journal has affected your reading. Therefore, I have modified Table 1 to present the complete content of the survey questionnaire, so that readers can better understand the measurement of brand reputation in this article. The following BR1-BR4 are measurement items for brand reputation.  The basis for selecting brand reputation as the school logo in this article is as follows.  Brand reputation is a concentrated reflection of consumer attitudes towards regional public brands, and is also considered the most important achievement in the construction of regional public brands (Juan et al., 2021). Moreover, the regional public brand of agricultural products, as a public asset within the region, mainly affects the sales of related enterprise products in the region through brand reputation (Yongdong, 2023).  BR1: Yingde black tea has a high brand awareness.  BR2: Yingde black tea has a unique brand image.  BR3: Yingde black tea has a good brand evaluation.  BR4: Yingde black tea has many loyal customers who repeat purchases. |
|  | 4. The hypotheses amony policy support, industrial cluster, resource endowment, and regional culture, as shown in Figure 1, should be well reconsidered. Will regional culture have an impact on policy support? Will the development of industrial cluster have an impact on related policy support? | The questions you raised are precisely the focus of this article's research. When conducting literature review in this article, it was found that there is inconsistency in the views of existing research results on the relationship between regional culture, government support, and industrial clusters. Therefore, the idea of conducting this study emerged.  The research findings of this article verify the mediating role of industrial clusters between government support, resource endowment, and the reputation of regional public brands for agricultural products. Regional culture plays a mediating role between government support, resource endowment, and the reputation of regional public brands for agricultural products.  However, whether regional culture and industrial clusters have a reverse effect on government support was not taken into account in the initial research of this article. This will be an issue that needs further in-depth attention when conducting further research in this article. Thank you very much for raising this issue.  Based on your suggestion, I have made some modifications to the research hypothesis section to strengthen the research foundation related to the mediation hypothesis, and added the following content.  The development of industrial clusters will further strengthen government support for this industry and introduce more favorable policies (Zheng Ailin, 2024). The government helps promote the construction of regional culture by organizing exhibitions, celebrations, and food culture festivals for characteristic agricultural products (Yue Ju, 2024). The prosperity of regional culture will also lead the government to increase investment and promotion in the construction of this culture (Wang Zhaofeng, 2024). |
|  | 5. All questions of the questionnaire are from literature? No new questions based on the proposed research were put forward? | Yes, all questions are sourced from existing literature survey questionnaires. However, before conducting formal large-scale data collection, this article randomly selected 65 respondents (13 government officials related to Yingde black tea, 14 staff members of the black tea association, 15 staff members of black tea enterprises, 6 staff members of the black tea research institute, and 17 black tea growers) for small sample testing, and deleted some non compliant questions based on the test results, For example, regarding government support, improving the entrepreneurial environment and guiding the integration of industry, academia, and research. |
|  | 6. Section 5 is mainly about the analysis and further discussion about statistical results, the theoretical and practical implications are not clearly stated. How can the proposed model and results drive theoretical research progress or guide industrial improvement? For instance, how to cultivate industrial cluster and regional culture? Which direction to guide? | Thank you very much for your suggestion. This time, the content of the theoretical contribution section has been revised to provide a clearer and more explicit expression of the theoretical contribution of this article, and it has been separately presented in the form of 6.2. This can make it easy for readers to understand the theoretical contribution of this article.  The following content has been added:  The mediating role of regional culture reveals the main pathways through which resource endowments play a role in the regional public branding of agricultural products. The intermediary role of industrial clusters reveals the main path through which government support plays a role in promoting regional public branding of agricultural products.  Therefore, the government supports the cultivation of industrial clusters as the main direction, while resource endowment is mainly based on regional culture. |
|  | 7. According to the practical implication of Section 5.2, the construction of a regional public brand does not only need policy support from the local government, but also financial support, then the construction process is local government-dominated or industry/enterprises-dominated? | Your feedback was very useful for revising this article and helped me identify an important error in the translation process. This article mistakenly translated "government support" as "policy support" during translation. This time, all "policy support" will be changed to "government support". In this study, government supported measurement items include: the impact of government on regional public brands of agricultural products in industrial policies, financial support, market supervision, strategic planning, and public services. The following are specific measurement items PS1-PS5 supported by the government. Therefore, this article also modified Table 1 to present the content of the survey questionnaire in detail, so that readers can better understand government support.  PS1: The Yingde City Government has issued policies to support the development of the Yingde black tea industry.  PS2: The Yingde City Government has invested financial funds to support the development of the Yingde black tea industry.  PS3: The Yingde City Government has strengthened supervision and protection of the black tea market.  PS4: The Yingde City Government has formulated a strategic plan for the development of the black tea industry.  PS5: The Yingde City Government has increased the construction of public services and facilities.  Therefore, the viewpoint of this article is that the construction of regional public brands for agricultural products requires government support in industrial policies, financial support, market supervision, strategic planning, and public services. This study found that the effect value of government support on the reputation of regional public brands of agricultural products through industrial clusters is greater than the effect value of government support on the reputation of regional public brands of agricultural products through regional culture, and regional public brands of agricultural products have the attribute of public goods. Therefore, the construction process of regional public brands for agricultural products is led by local governments.  Based on the research results, modifications have been made to the practical implications, making their content more clear and reasonable. |
