# Supplementary material for: A structural equation model to access the regional public brands of agricultural products: Case of Chinese Yingde black tea
Source: PLoS One. 2024 Sep 27;19(9):e0310722. doi: 10.1371/journal.pone.0310722 (PMC11432859; doi:10.1371/journal.pone.0310722)
Supplement: Attachment — Submitted filename: Response to Reviewers.doc [file pone.0310722.s004.doc]

**Response to Reviewers**

Dear Reviewers,

We sincerely appreciate the valuable suggestions you have provided for improving our manuscript. These recommendations are of great importance to the refinement of our work. In response, we have carefully revised the manuscript according to all the suggestions. The specific changes made are detailed below.

| **Reviewer** | **Recommendation** | **Modification status** |
| --- | --- | --- |
| Journal Requirements | Please review your reference list to ensure that it is complete and correct. If you have cited papers that have been retracted, please include the rationale for doing so in the manuscript text, or remove these references and replace them with relevant current references. Any changes to the reference list should be mentioned in the rebuttal letter that accompanies your revised manuscript. If you need to cite a retracted article, indicate the article’s retracted status in the References list and also include a citation and full reference for the retraction notice. | We have reviewed all the references cited in the manuscript and found no evidence of any retracted publications. |
| Reviewer #1 | The authors have addressed most of my observations. However, point 3 is not satisfied. I am not asking to include socio-psychological drivers in this research. What I ask, is to add a small section explaining the role of socio-psychological drivers in this topic, and also explain that one of the limitations of the study is that these drivers were not included. Please add an argument for this omission. | Your suggestion is excellent. Through our literature review, we have indeed found that some scholars have explored regional public brands of agricultural products from a social psychology perspective. Based on this, we have added the following content to the manuscript.  **(1) In the literature review section, we have added a discussion on the influence of social psychological factors on regional public brands of agricultural products and explained the reasons for not including them in this study, as detailed below.**  Some scholars have explored the relationship between individuals and regional public brands of agricultural products from a social psychology perspective. Multifaceted interactions have a significant positive impact on farmers' willingness to participate in the co-creation of regional public brands for agricultural products, with psychological contracts serving as a mediating factor between these interactions and farmers' co-creation intentions(Juan, 2021). There is a close relationship between regional public brands of agricultural products and consumers' emotional attitudes, with both exerting mutual influence on each other(Fuyin, 2024). The greater the psychological distance between consumers and agricultural products, the higher consumers rate those products from that region(Li Xia, 2022). Jian, D. et al. explored farmers' willingness to protect the agricultural ecosystem from a social psychology perspective, based on the Theory of Planned Behavior. They found that farmers' behavior is significantly influenced by their willingness to protect the ecosystem, which in turn is significantly affected by their attitudes, subjective norms, and perceived behavioral control(Deng J, 2016). The psychological factors of farmers pertain to the micro-level individual psychological changes(Cheng Hong, 2023); however, this study primarily explores the mechanisms of regional public brand development for agricultural products from a macro-level perspective. Therefore, this study does not incorporate social psychological factors into the research process.  **(2) In the limitations section, we addressed the omission of social psychological factors, as detailed below.**  Again, this study primarily explores the mechanisms of regional public brand development for agricultural products from a macro-level perspective, without incorporating micro-level individual psychological factors into the research. However, farmers' willingness to participate in the co-creation of regional public brands for agricultural products, as well as consumers' emotional interactions with these brands, both have an impact on the development of regional public brands for agricultural products.  **(3) In the future research section, we outlined plans for further exploring regional public brands of agricultural products from a social psychology perspective, as detailed below.**  Finally, the study will explore the specific impacts of individual psychological factors from a social psychology perspective on the process of developing regional public brands for agricultural products, aiming to further deepen the theoretical understanding of these brands. |
